# Rhizosphere priming: a nutrient perspective

**DOI:** 10.3389/fmicb.2013.00216

**Published:** 2013-07-29

**Authors:** Feike A. Dijkstra, Yolima Carrillo, Elise Pendall, Jack A. Morgan

**Affiliations:** ^1^Department of Environmental Sciences, Centre for Carbon, Water, and Food, The University of SydneyCamden, NSW, Australia; ^2^Department of Botany and Program in Ecology, University of WyomingLaramie, WY, USA; ^3^Rangeland Resources Research Unit, USDA-ARSFort Collins, CO, USA

**Keywords:** ^15^N tracer, microbial mining, N:P stoichiometry, nutrient competition, preferential substrate utilization, progressive nitrogen limitation, root exudates

## Abstract

Rhizosphere priming is the change in decomposition of soil organic matter (SOM) caused by root activity. Rhizosphere priming plays a crucial role in soil carbon (C) dynamics and their response to global climate change. Rhizosphere priming may be affected by soil nutrient availability, but rhizosphere priming itself can also affect nutrient supply to plants. These interactive effects may be of particular relevance in understanding the sustained increase in plant growth and nutrient supply in response to a rise in atmospheric CO_2_ concentration. We examined how these interactions were affected by elevated CO_2_ in two similar semiarid grassland field studies. We found that an increase in rhizosphere priming enhanced the release of nitrogen (N) through decomposition of a larger fraction of SOM in one study, but not in the other. We postulate that rhizosphere priming may enhance N supply to plants in systems that are N limited, but that rhizosphere priming may not occur in systems that are phosphorus (P) limited. Under P limitation, rhizodeposition may be used for mobilization of P, rather than for decomposition of SOM. Therefore, with increasing atmospheric CO_2_ concentrations, rhizosphere priming may play a larger role in affecting C sequestration in N poor than in P poor soils.

## Introduction

Rhizosphere priming is the change in soil organic matter (SOM) decomposition caused by plant root activity that is often associated with rhizodeposition (Kuzyakov, 2002). A substantial fraction of net carbon assimilation goes into the soil as rhizodeposition. Estimates of how much C is allocated to rhizodeposition vary widely among plant species, with plant age, soil type, and nutrient availability, and are on average between 11 and 17% of net fixed C (Nguyen, 2003; Jones et al., 2009). Rhizodeposition is an important energy source for the microbial production of extra-cellular enzymes that break down SOM (Schimel and Weintraub, 2003; Blagodatskaya and Kuzyakov, 2008; Averill and Finzi, 2011). The subsequent change in SOM decomposition (i.e., the rhizosphere priming effect) is usually measured by comparing the CO_2_ produced from SOM in a soil with and without plants. Often, CO_2_ produced from SOM in planted soil is greater than in the unplanted or fallow soil, and is referred to as a positive priming effect (Kuzyakov, 2002). However, smaller CO_2_ production in planted compared to unplanted soil, or a negative priming effect, has also been observed (Cheng, 1996; Bader and Cheng, 2007). Although rhizosphere priming effects have frequently been reported in a variety of soil-plant systems, the mechanisms behind these effects remain unclear (Kuzyakov, 2010).

## Effects of soil nutrient availability on rhizosphere priming

**Figure 1:**
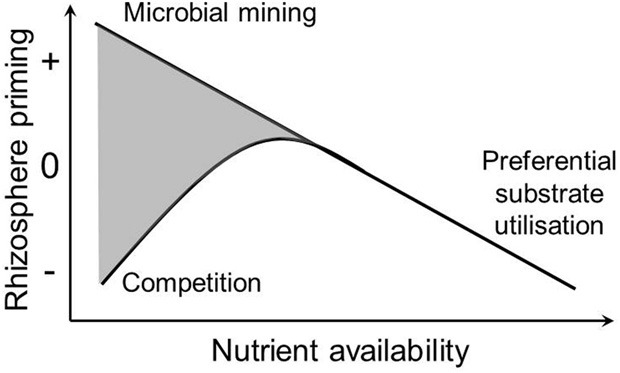
**Hypothetical relationship between soil nutrient availability and rhizosphere priming**. Three nutrient-centered hypotheses are illustrated: Microbial mining: microbes utilize rhizodeposition to mine for nutrients in SOM thereby causing a positive rhizosphere priming effect when nutrient availability is low; Preferential substrate utilization: microbes switch from decomposing SOM to utilizing rhizodeposition when nutrient availability is high; Competition: microbes compete for nutrients with plants causing a negative rhizosphere priming effect because microbial growth and decomposition are nutrient limited. Both positive and negative rhizosphere priming can occur under low nutrient availability (gray area).

The direction and magnitude of rhizosphere priming have been related to soil nutrient availability. Plants and microbes require C and nutrients within specific boundaries and at the same time affect the relative availability of C and nutrients in their immediate environment, the rhizosphere. Because of this close interdependence between C and nutrients, the nutrient status of the soil is an important factor for rhizosphere priming. Several hypotheses have been proposed explaining the relationship between rhizosphere priming and soil nutrient availability (Figure 1). First, in soils of low nutrient availability, inputs of energy-rich carbon compounds from roots may be used for the production of extra-cellular enzymes that can release nutrients locked in SOM (Asmar et al., 1994; Brzostek et al., 2012). Under these conditions, microbes may use root exudates to release nutrients thereby meeting their nutrient requirement. This has also been referred to as the microbial mining hypothesis (Craine et al., 2007; Fontaine et al., 2011). Because rhizosphere priming effects are usually measured through changes in CO_2_ production, microbial mining for nutrients associated with rhizosphere priming should only relate to nutrients released through oxidation of SOM accompanied by the production CO_2_. Much of the N in humified SOM is released through oxidation (biological mineralization). While some organic N compounds (proteins, amino acids, amino sugars) do not need to be oxidized for the N to be utilized, the C skeletons of these compounds are often catabolized by microbes thereby producing CO_2_. On the other hand, organic P is mostly released through hydrolysis without CO_2_ production (biochemical mineralization, McGill and Cole, 1981). Therefore, the microbial mining hypothesis may be more important for N than for P.

Second, in soils of high nutrient availability, a negative priming effect may occur. Under conditions of high nutrient availability, there is less need for microbes to mine nutrients, but instead, microbes may switch from decomposing recalcitrant SOM to utilizing labile root exudates for their carbon and energy requirements (Blagodatskaya et al., 2007; Guenet et al., 2010). As a result, decomposition of SOM could decrease with inputs of root exudates. This has been referred to as the preferential substrate utilization hypothesis (Cheng, 1999) (Figure 1).

A third nutrient-centered mechanism that has been proposed for explaining negative rhizosphere priming effects is when plants and microbes compete for the same nutrients. When plants remove nutrients from the soil through uptake they may reduce microbial decomposition (Dijkstra et al., 2010b; Pausch et al., 2013). The resulting negative rhizosphere priming effect may be stronger when nutrient availability is already low and limiting both plant and microbial growth (competition hypothesis, Cheng, 1999) (Figure 1). Production of rhizodeposits may also be a strategy for slow-growing plant species to lower soil N availability thereby outcompeting neighboring fast-growing plant species (Meier et al., 2009).

Low soil nutrient conditions can invoke both positive and negative rhizosphere priming (Figure 1). Clearly, there is a need to better understand why rhizodeposition in soils with low nutrient conditions sometimes result in enhanced microbial mining for nutrients (and a positive rhizosphere priming effect) and at other times in enhanced competition for nutrients inducing reduced microbial activity (and a negative rhizosphere priming effect). Several explanations may be involved in observations of positive and negative priming effects, including soil microbial community effects, quality and stoichiometry of the root exudates, and the relative availability of N and P, while none of these explanations are mutually exclusive.

First, dominance of one group of microbes over the other could potentially determine whether rhizodeposition results in negative or positive rhizosphere priming under low nutrient conditions. Microbes vary tremendously in their ability to decompose SOM. While rhizodeposition may primarily increase growth and activity of fast growing microbes (r-strategists), a proportion of the rhizodeposition may be utilized by slow growing microbes decomposing recalcitrant organic matter (K-strategists), particularly when nutrient availability is low (Fontaine et al., 2003). Fungi, gram-negative and gram-positive bacteria have all been associated with enhanced SOM decomposition with increased rhizodeposition and input of other labile C (Nottingham et al., 2009; Bird et al., 2011; Fontaine et al., 2011; Garcia-Pausas and Paterson, 2011). Bacteria may be more sensitive to competition for nutrients with plants than fungi because fungal hyphae have a greater ability to explore the soil (Otten et al., 2001), and therefore fungi may escape competition for nutrients with plants. Furthermore, mycorrhizae, a special group of fungi that grow in direct association with plants, may supply nutrients directly to plants in return for plant C, thereby reducing nutrient competition between plants and mycorrhizae (Koide, 1991).

If the soil microbial community is dominated by bacteria that are activated close to the root, then the microbial competition hypothesis (or preferential substrate utilization hypothesis under high nutrient availability) may prevail resulting in negative rhizosphere priming. These bacteria would be dominated by r-strategists and utilize fresh exudates (Dorodnikov et al., 2009). On the other hand, if the soil microbial community is dominated by fungi, supply of plant C may stimulate fungi to mine for nutrients further away from the roots resulting in positive rhizosphere priming. These fungal decomposers act more like K-strategists, and may be saprotrophic or mycorrhizal (Talbot et al., 2008).

Second, the direction and magnitude of rhizosphere priming under low nutrient conditions may depend on the type of organic compounds released by plants. Plant roots release a myriad of organic compounds, not only from root exudation, but also from mucilage, as sloughed cells via mechanical abrasion, and from root death (Jones et al., 2004, 2009). Because these compounds have different stoichiometric and energetic properties, rhizodeposition may have variable effects on priming (Mary et al., 1993; Hamer and Marschner, 2005; Kuzyakov and Bol, 2006). Although many of these compounds often showed idiosyncratic priming effects, root exudates that generate more alkalinity during their decomposition or contain more N have been shown to cause greater priming (Rukshana et al., 2012; Drake et al., 2013).

Third, contrasting rhizosphere priming effects under low nutrient availability may occur because priming also depends on soil properties such as total C content and texture (Zhang et al., 2013), mineralogy (Rasmussen et al., 2007), pH (Blagodatskaya and Kuzyakov, 2008; Luo et al., 2011), and heavy metal concentration (Ohm et al., 2011). Here we propose that the contrasting effects of rhizosphere priming under low nutrient availability can also be related to the relative availability of N and P in the soil.

## N and P cycling and their role in rhizosphere priming

As discussed above, the supply of N to plants and microbes in soils predominantly occurs through oxidation of organic matter whereby N is mineralized. Phosphorus can also be released from SOM, but in soils with low organic P, inorganic sources are an important source for P supply (Walker and Syers, 1976). For instance, in calcareous soils much of the soil P is contained in calcium phosphates and the supply of P is regulated by precipitation/dissolution equilibria with P in soil solution (Lajtha and Bloomer, 1988; Tunesi et al., 1999). Similarly, in many acidic soils, P is bound to Al and Fe oxides, and the supply of P to plants is controlled by adsorption/desorption processes (Sanyal and Datta, 1991). Furthermore, much of the organic P is present in soil as monoesters and diesters (Doolette and Smernik, 2011). The P in these bonds can be released by hydrolysis with the help of phosphatase enzymes (without causing CO_2_ production, Nannipieri et al., 2011), rather than through oxidation of organic matter (causing CO_2_ production). Therefore, the supply of N and P to plants is decoupled in many soil types (McGill and Cole, 1981).

Terrestrial ecosystems are frequently limited by P (Elser et al., 2007; Harpole et al., 2011). Microbes in particular have a high P requirement relative to N, where microbial N:P ratios are often lower than the plant or SOM N:P ratios from the same system (Cleveland and Liptzin, 2007). Microbial activity and growth can be limited by P, which has mostly been observed in highly weathered tropical soils (Cleveland et al., 2002; Ehlers et al., 2010), but also in a calcareous (Raiesi and Ghollarata, 2006), peat (Amador and Jones, 1993), and boreal forest soils (Giesler et al., 2002).

**Figure 2:**
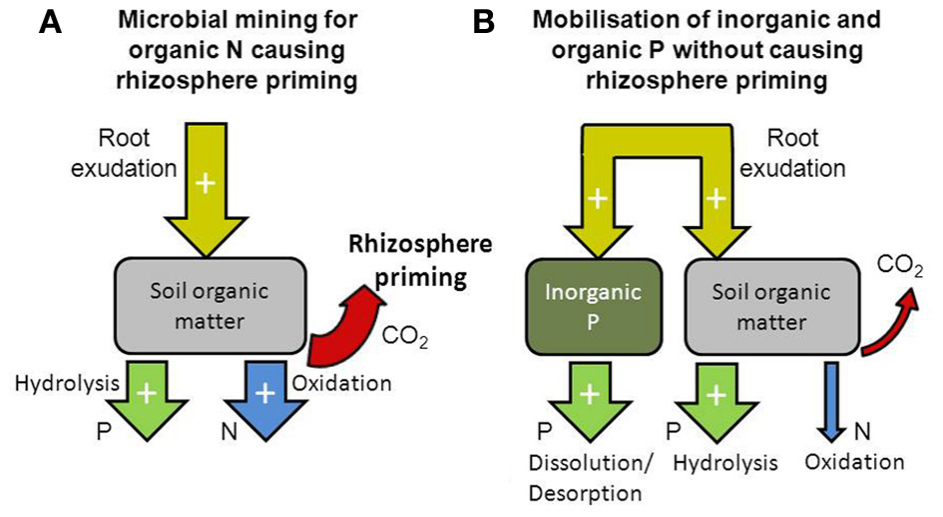
**Diagram illustrating how the availability of nitrogen and phosphorus in the soil can influence rhizosphere priming**. When nitrogen availability is low, microbes utilize rhizodeposition to mine for nitrogen locked in organic matter thereby increasing rhizosphere priming and release of nitrogen (through oxidation of SOM) and phosphorus [mostly through hydrolysis of P-esters in SOM, **(A)**]. When phosphorus availability is low, rhizodeposition is utilized to mobilize phosphorus from inorganic and organic sources (through dissolution/desorption and hydrolysis, respectively) thereby increasing the release of phosphorus without affecting rhizosphere priming **(B)**.

Whether the input of C compounds via rhizodeposition results in altered SOM decomposition may depend on whether microbial activity is N or P limited. In soils where microbes are more limited by N than by P, root exudates could be utilized to mine for N through enhanced SOM decomposition. On the other hand, in soils where microbes are more limited by P than by N, root exudates are not needed by microbes for releasing N from SOM, but instead, could be used to mobilize P from inorganic or organic sources. Root exudates could be utilized by microbes to produce phosphatase extracellular enzymes releasing P through hydrolysis (Dakora and Phillips, 2002). Increased levels of microbial biomass and phosphatase extracellular enzymes have been observed in the rhizosphere (Chen et al., 2002), but it is unclear to what degree phosphatase extracellular enzymes are produced by plants or microbes (George et al., 2011). Root exudates can also directly increase P mobilization by increasing desorption and solubilisation from mineral surfaces through ligand exchange and dissolution (Dakora and Phillips, 2002; George et al., 2011). We hypothesise that microbial N limitation results in rhizosphere priming, while microbial P limitation does not (Figure 2). After discussing the effects of rhizosphere priming on nutrient availability, we will illustrate this hypothesis with examples of rhizosphere priming effects that were observed under elevated atmospheric CO_2_ concentration.

## Rhizosphere priming effects on nutrient availability

While some research has been done on how rhizosphere priming is affected by nutrient availability, particularly N availability, considerably less is known regarding the consequences of rhizosphere priming for nutrient cycling and plant nutrient uptake. From an evolutionary perspective it could be argued that the loss of expensive energy-rich carbon compounds into the soil through root exudation should result in some benefit to the plant. Does the stimulation of SOM decomposition through rhizosphere priming result in increased nutrient availability to plants or does it only result in increased microbial nutrient immobilization? There are only a limited number of studies that have tried to tackle this question.

Positive priming effects should be associated with net N mineralization, but Cheng (2009) observed that the amount of net mineral N released from the accelerated SOM decomposition associated with the rhizosphere priming effect was much lower than the expected amount based on the C:N ratio of the SOM. This suggests that much of the N associated with the increase in SOM decomposition remained in the microbial biomass or was returned to the organic soil pool. On the other hand, a range of positive rhizosphere priming effects were observed among six different soil type-plant species combinations that also resulted in substantial increases in net N mineralization (Dijkstra et al., 2009). Except for one soil-plant combination, rhizosphere priming effects were positively related to gross N mineralization and plant N uptake, suggesting that rhizosphere priming not only enhanced microbial mining for N, but also enhanced the release of N for plant uptake. An increase in net N mineralization with root exudation could also occur because of microbial grazing by protozoa (Clarholm, 1985) creating a microbial loop. According to this microbial loop hypothesis, increased microbial growth in response to root exudation causes increased microbial immobilization of N, which in return is released for plant uptake after grazing by protozoa or nematodes.

However, there appears to be a fine balance between how rhizosphere priming affects microbial mineralization and immobilization. Microbial immobilization increased more than gross N mineralization with increased root exudation of three species of tree seedlings so that net N mineralization was reduced at high rates of root exudation (Bengtson et al., 2012). In modeling and field experiments Drake et al. (2013), simulated exudates and showed that adding C alone enhanced SOM decomposition, but adding C and N together stimulated SOM decomposition and N mineralization significantly more than C alone. These results suggest that both quantity and quality of root exudation have important consequences for the release of N through rhizosphere priming.

## Rhizosphere priming under elevated atmospheric [CO_2_]

Atmospheric CO_2_ concentrations have increased by more than 35% during the last 150 years and will continue to rise in the future (Forster et al., 2007), causing large impacts on C cycling in terrestrial ecosystems (Heimann and Reichstein, 2008). The immediate plant response to an increase in atmospheric CO_2_ is often an increase in photosynthesis and net primary production (Amthor, 1995). Several studies have indicated that elevated CO_2_ also increases rhizodeposition (Darrah, 1996; Pendall et al., 2004; Fransson and Johansson, 2010), and could potentially increase rhizosphere priming. Indeed, increased loss of soil C or mineral-associated organic matter under elevated CO_2_ has been associated with greater rhizosphere priming (Carney et al., 2007; Hofmockel et al., 2011b).

An increase in SOM decomposition caused by rhizosphere priming under elevated CO_2_ may also affect N cycling, and this has important ramifications for long-term responses of terrestrial ecosystems to elevated CO_2_. Plant growth in most terrestrial ecosystems is N limited (Vitousek and Howarth, 1991). It has been suggested that without external input of N, elevated CO_2_ will reduce N availability to plants in the long-term and that therefore plant growth responses to elevated CO_2_ cannot be sustained (Luo et al., 2004). A crucial component of this concept of Progressive N Limitation (PNL) is the expectation that N availability is reduced under elevated CO_2_ because of increased plant N uptake and immobilization in long-lived plant biomass, and because of increased microbial N immobilization associated with increased C inputs into the soil. Indeed, elevated CO_2_ often reduces N availability in the soil (Díaz et al., 1993; Gill et al., 2002; Reich et al., 2006). Tree growth in a temperate forest increased during the first 6 years in response to elevated CO_2_, but this effect disappeared after 11 years (Norby et al., 2010). It was suggested that a decline in soil N availability constrained the plant growth responses to elevated CO_2_ in the long-term thereby providing support for the PNL concept.

However, PNL has not always been observed (Luo et al., 2006) and rhizosphere priming may be one of the mechanisms responsible for the lack of PNL. Tree growth in a temperate forest in North Carolina was still higher after 10 years of elevated CO_2_ (McCarthy et al., 2010). Moreover, plant N uptake remained higher under elevated CO_2_, which appears to have caused the sustained increase in tree growth in response to elevated CO_2_ in this study (Drake et al., 2011). While some of this extra N may have been taken up from deeper soil layers (Finzi et al., 2006), it was also shown that elevated CO_2_ enhanced root exudation and N release from SOM decomposition through rhizosphere priming (Phillips et al., 2011, 2012) Increased rhizosphere priming and plant N uptake under elevated CO_2_ has also been observed in several other studies (Martín-Olmedo et al., 2002; de Graaff et al., 2009; Hofmockel et al., 2011a). These results suggest that enhanced rhizosphere priming could delay or at least alleviate PNL under elevated CO_2_.

## Contrasting elevated CO_2_ effects on N cycling in semiarid grasslands

**Figure 3:**
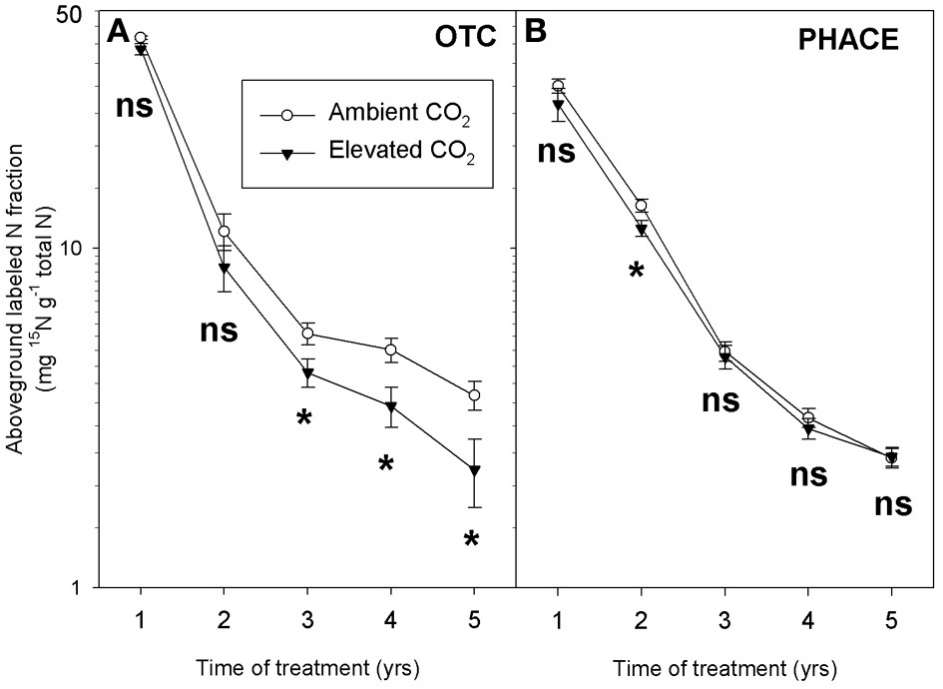
**Labeled N fractions (expressed per total amount of N) in aboveground biomass over time in the OTC (A) and PHACE (B) experiment**. A ^15^N label was added to the soil in the Spring of year 1 and aboveground biomass was sampled at peak biomass in July in the following 5 years. For each year, CO_2_ treatment effects were tested with ANOVA (ns: not significant, ^*^P < 0.05).

Sustained increases in N cycling and plant N uptake under elevated CO_2_ were also observed in a semiarid grassland in Colorado, USA (King et al., 2004; Dijkstra et al., 2008). Enhanced N cycling and microbial mining for N under elevated CO_2_ was illustrated with a ^15^N tracer study. In this field experiment ^15^N (as ^15^NH_4_^15^NO_3_) was added as a tracer to the soil and followed into aboveground plant biomass collected in plots exposed to ambient and elevated CO_2_ using open top chambers, up to 5 years after the pulse addition (OTC experiment). The ^15^N label in plant tissue, expressed as a fraction of total aboveground plant tissue N, decreased with time (Dijkstra et al., 2008) (Figure 3A). The decrease of the ^15^N label was explained by ongoing mineralization of unlabeled N in the soil that progressively diluted the ^15^N label in the available N pool to plants (Dijkstra, 2009). This dilution of the ^15^N label in the plant was faster under elevated CO_2_, suggesting that mineralization of unlabeled N from SOM was enhanced under elevated CO_2_. Rhizodeposition was also greater under elevated CO_2_ in this system (Pendall et al., 2004). These combined results suggest that elevated CO_2_ may have increased rhizosphere priming through microbial mining for N and subsequent release of N for plant uptake.

We conducted a similar experiment in the Prairie Heating And CO_2_ Enrichment (PHACE) experiment in Wyoming, USA (Dijkstra et al., 2010a; Morgan et al., 2011). The northern mixed prairie vegetation of the PHACE experiment is similar to that of the shortgrass steppe of the OTC experiment in Colorado with *Bouteloua gracilis* (a warm season C4 grass), *Pascopyrum smithii* and *Hesperostipa comata* (two cool-season C3 grasses) being the dominant species comprising 80% or more of the aboveground biomass at both sites, although net primary productivity is greater at PHACE than at OTC. Free Air CO_2_ Enrichment technology was used in the PHACE experiment to increase the CO_2_ concentration to 600 ppm, which is lower than the elevated CO_2_ treatment in the OTC experiment (720 ppm). Furthermore, a warming treatment (1.5/3°C above ambient during the day/night) using infrared heaters was included in the PHACE experiment in a full factorial design.

As in the OTC experiment, we added a ^15^N tracer to the plots and followed the ^15^N label into aboveground biomass during the following years. The ^15^N label was added by spraying a K^15^NO_3_ solution (99 atom% ^15^N) onto the plots in 2007. As in the OTC experiment the ^15^N label, expressed as a fraction of the total plant N, decreased with time due to dilution of the label with non-labeled N from mineralization in the soil. However, in contrast to the OTC experiment, the decrease of the ^15^N label in aboveground biomass was not enhanced under elevated CO_2_ (Figure 3B). These results suggest that elevated CO_2_ did not enhance microbial mining and release of unlabeled N in the PHACE experiment.

## Does rhizosphere priming under elevated CO_2_ depend on relative availability of N and P?

**Figure 4:**
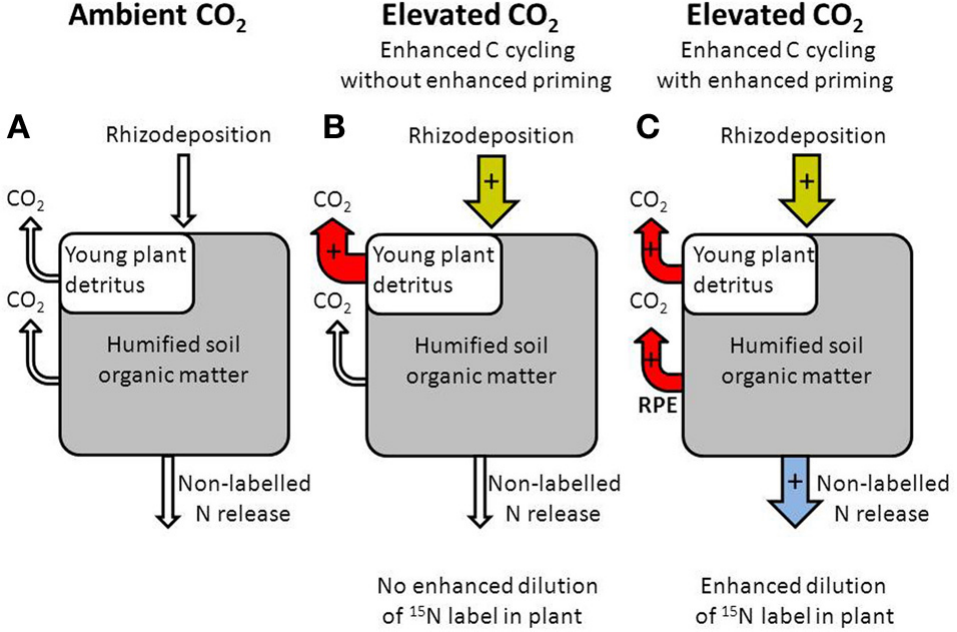
**Diagram illustrating the role of rhizodeposition for carbon cycling under ambient (A) and elevated CO_2_ (B,C)**. An increase in rhizodeposition under elevated CO_2_ can enhance the decomposition of young plant detritus only without affecting the decomposition of humified SOM and without affecting the release of non-labeled nitrogen **(B)**, or can enhance the decomposition of plant detritus and humified SOM (rhizosphere priming effect or RPE) thereby increasing release of non-labeled nitrogen **(C)**.

Why did these similar semiarid grasslands respond differently to elevated CO_2_ in terms of its effect on N cycling? A possible explanation is that in the PHACE experiment elevated CO_2_ did not increase rhizosphere priming of SOM. However, elevated CO_2_ resulted in larger labile soil C pools, although not in all years (Carrillo et al., 2011). Further, elevated CO_2_ increased rates of heterotrophic respiration in the PHACE experiment (Pendall et al., 2013). These results suggest that elevated CO_2_ may have enhanced rhizosphere priming of native soil organic C. However, it is also possible that elevated CO_2_ enhanced input and cycling of the new and relatively labile soil C only without affecting decomposition of the native soil C pool (e.g., Hungate et al., 1997) (Figure 4). If elevated CO_2_ increased the cycling of new C without affecting decomposition of native soil organic C, this would not result in enhanced dilution of the ^15^N in the plant (Dijkstra, 2009). Only an increase in the decomposition of native soil organic C (due to rhizosphere priming) would result in enhanced dilution of the ^15^N label in the plant. Interestingly, (Allard et al., 2006) also found no enhanced plant N uptake under elevated CO_2_ despite enhanced C cycling.

Why then would greater inputs of labile C under elevated CO_2_ result in enhanced rhizosphere priming of native soil organic C in the OTC experiment, but only result in enhanced cycling of labile C in the PHACE experiment? We postulate that these contrasting effects of elevated CO_2_ on soil C cycling may have occurred because of differences in the availability and cycling of N and P between the two sites.

As explained above, under conditions of low N availability, root exudates could be used by microbes to improve N supply by enhancing the decomposition of recalcitrant SOM that is relatively rich in N (microbial mining hypothesis), possibly with an extra supply of N-degrading enzymes (Drake et al., 2013). However, as described above, root exudates could also be used for the production of phosphatase extracellular enzymes hydrolyzing organic P (by plants and microbes) and to increase mobilization of P (Dakora and Phillips, 2002; George et al., 2011) without affecting SOM decomposition.

In the PHACE experiment we observed that the availability and plant uptake of P compared to that of N increased under elevated CO_2_ (Dijkstra et al., 2012). The PHACE experiment was conducted on a calcareous soil high in insoluble calcium phosphates (41% of total soil P was in inorganic form, Dijkstra et al., 2012) that are not directly available to plants. The fixation of P as calcium phosphates may have caused low P availability possibly limiting microbial activity and plant growth. An increase in root exudation under elevated CO_2_ in this system may have increased P dissolution and mobilization without affecting net N release from native SOM. We have limited data on the availability of P compared to N in the soil of the OTC experiment. While soil P availability was similarly low at both sites (between 4 and 11 mg P kg^−1^ soil in the OTC experiment and between 4 and 7 mg P kg^−1^ soil in the PHACE experiment using 0.5 M NaHCO_3_ extractions), a semiarid grassland similar to the grassland used in the OTC experiment strongly responded to N fertilization (Lauenroth et al., 1978). This suggests that plants and microbes may have been more limited by N than by P in the OTC experiment.

We propose that contrasting effects of elevated CO_2_ on ^15^N dilution in plant biomass in the OTC and PHACE experiments were due to differences in N and P availability to microbial activity and plant growth. In the N limited semiarid grassland, where the OTC experiment was conducted (Lauenroth et al., 1978), increased root exudation under elevated CO_2_ resulted in a greater rhizosphere priming thereby enhancing SOM decomposition and mineralization of N, and possibly P. The enhanced N mineralization from native SOM then resulted in enhanced dilution of the ^15^N label in the plant (Figure 2). On the other hand, in the PHACE experiment where P availability was low (Dijkstra et al., 2012) and that may have limited microbial activity and plant growth more than N, the increase in root exudation under elevated CO_2_ may have increased the dissolution/desorption and mobilization of P, more so than enhancing decomposition of native SOM. As a result, the dilution of the ^15^N label in aboveground plant biomass with time was unaffected by elevated CO_2_. Others have also suggested that enhanced rhizodeposition under elevated CO_2_ may be utilized for mobilizing P, rather than for enhancing SOM decomposition (Cardon, 1996; Lloyd et al., 2001).

## Conclusion

Several studies have suggested that elevated CO_2_ can enhance SOM decomposition through increased rhizosphere priming effects (Cheng, 1999; Paterson et al., 2008; Phillips et al., 2011). An increase in rhizosphere priming has important implications for long-term C sequestration in soils under elevated CO_2_ and how this feedbacks to the global climate. However, the magnitude and direction of the rhizosphere priming effect may strongly depend on the relative availability of N and P in the soil (Bradford et al., 2008; Milcu et al., 2011; Sullivan and Hart, 2013). Although rhizosphere priming is influenced by several factors, we argue that the relative availability of N and P has to be considered in understanding how perturbations such as climate change affect rhizosphere priming and soil C sequestration.

### Conflict of interest statement

The authors declare that the research was conducted in the absence of any commercial or financial relationships that could be construed as a potential conflict of interest.
